# Association between Fecal Microbiota, SCFA, Gut Integrity Markers and Depressive Symptoms in Patients Treated in the Past with Bariatric Surgery—The Cross-Sectional Study

**DOI:** 10.3390/nu14245372

**Published:** 2022-12-17

**Authors:** Natalia Komorniak, Alexandra Martynova-Van Kley, Armen Nalian, Michał Wroński, Krzysztof Kaseja, Bartosz Kowalewski, Karolina Kaźmierczak-Siedlecka, Igor Łoniewski, Mariusz Kaczmarczyk, Konrad Podsiadło, Paweł Bogdański, Joanna Palma, Ewa Stachowska

**Affiliations:** 1 Department of Human Nutrition and Metabolomics, Pomeranian Medical University in Szczecin, 71-460 Szczecin, Poland; 2 Department of Biology, Stephen F. Austin State University, Nacogdoches, TX 75962, USA; 3 Department of Psychiatry, Pomeranian Medical University in Szczecin, 71-460 Szczecin, Poland; 4 Department of General Surgery and Transplantation, Pomeranian Medical University in Szczecin, 70-111 Szczecin, Poland; 5 Independent Provincial Public Hospital Complex in Szczecin-Zdunowo, 70-891 Szczecin, Poland; 6 Department of Medical Laboratory Diagnostics, Farhenheit Biobank BBMRI.pl. 80-214 Gdańsk, Poland; 7 Sanprobi Sp.z.o.o. Sp.K., Kurza Stopka 5/C, 70-535 Szczecin, Poland; 8 Department of Treatment of Obesity, Metabolic Disorders and Clinical Dietetics, Poznan University of Medical Sciences, 60-569 Poznan, Poland; 9 Department of Biochemical Sciences, Pomeranian Medical University in Szczecin, 71-460 Szczecin, Poland

**Keywords:** bariatric surgery, depressive disorders, microbiome, Roux-en-Y gastric bypass, sleeve gastrectomy, depression, Beck scale, lipopolysaccharide, diet, zonulin

## Abstract

(1) Background: Depressive symptoms often appear after surgical treatment. (2) Methods: We involved 41 adults who underwent bariatric surgery a minimum of 6 months before the study and had the Beck scale ≥12. We analysed patients’ mental state, gut barrier markers, faecal short chain fatty acids, and microbiota. (3) Results: Gut microbiota composition differed significantly among patients undergoing two different types of surgery (F = 1.64, *p* = 0.00002). Additionally, we discovered an association between short chain fatty acids and the Beck scale (F = 1.22, *p* = 0.058). The rearrangement of bacterial metabolites may be due to the patients’ use of increased dietary protein, with insufficient intake of products containing vegetable fiber (Diet Quality Index (DQI-I )adequacy 22.55 (±3.46) points). (4) Conclusions: Bariatric surgery affects the gut microbiota, which may play an important role in the development of depressive and gastrointestinal symptoms in patients after bariatric surgery. Low fiber consumption and increased levels of faecal isobutyric acid may lead to intestinal inflammation. There is a need for further research on this topic including a larger sample size.

## 1. Introduction

The term microbiota-gut-brain axis refers to a network of connections involving many biological systems that enable two-way communication between intestinal microorganisms and the brain [1]. This combination is crucial for maintaining the homeostasis of the central nervous system and the digestive system as well as gut microbiota [1]. This two-way communication takes place via the vagus nerve and through influence on the immune system, the hypothalamic-pituitary-adrenal axis, and the metabolism of tryptophan, along with the ability of the microbiota to synthesize a number of neurotransmitters and short-chain fatty acids (SCFA) with neuroactive properties [2].

Bariatric surgery seems to be the optimal treatment for patients suffering from morbid obesity, allowing the patient to obtain weight reduction. It also provides metabolic effects and can provide relief from a number of diseases (e.g., hypertension or diabetes). Additionally, bariatric surgery may improve quality of life and extend the term of life [3]. The most frequently performed bariatric procedures include sleeve gastrectomy (SG) (61.4%) and Roux-en-Y gastric bypass (RYGB) (17%) [4].

The exact mechanisms inducing changes in the gut microbiota after certain types of bariatric procedures have not been closely examined. Factors influencing the composition of the gut microbiota after surgery include changes in the diet and nutritional status, differences in the anatomy and pH of the gastrointestinal tract, and variable gastrointestinal transit time as well as bile acid metabolism [5]. Microbiological alterations are usually described within the first 3 months after the operation [6,7,8]. These changes include an increase in the amount of *Akkermansia*, *Escherichia*, and *Klebsiella* and a decrease in the amount of *Lactobacillus*, *Bifidobacterium* or *Faecalibacterium prausnitzii,* and *Coprococcus comes* [9].

Notably, depressive symptoms decrease in the majority of patients within 6, 12, and 24 months after bariatric surgery. However, an increase in depressive and anxiety symptoms is observed after 36, 48, and 60 months [10].

Numerous studies confirm that mood is influenced by the composition of the gut microbiota [11]. The occurrence of mental disorders (including depression) is associated with the gut dysbiosis [12]. Among patients with major depressive disorders, compared to the control group, the decreased amount of *Firmicutes* and increased amount of *Bacteroidetes* is noted, resulting in reduced SCFA production and deterioration of the intestinal barrier function [13,14]. In this group of patients, an increased amount of *Actinobacteria*, *Enterobacteriaceae,* and *Alistipes* as well as decreased amounts of *Faecalibacterium* were also observed. Moreover, the occurrence of *Faecalibacterium* is negatively correlated with the severity of depressive symptoms, while *Prevotella* and *Klebsiella* correlate positively with this phenomenon. Additionally, among patients with major depressive disorders (MDD), a decrease in the amount of *Bifidobacterium* and *Lactobacillus* is observed [15].

The primary aims of the study were to assess the gut microbiota, SCFA, and markers of intestinal barrier integrity among patients ≥6 months after bariatric surgery who suffered from depressive symptoms. The secondary aims of the study included analysis of gastrointestinal symptoms (as abdominal pain, bloating, heartburn, nausea, bowel movement problems, diarrhea, and constipation), mental state, and diet. The tested hypothesis was that gut microbiota, its metabolites, and gut barrier integrity are associated with depressive symptoms.

## 2. Materials and Methods

**Figure 1:**
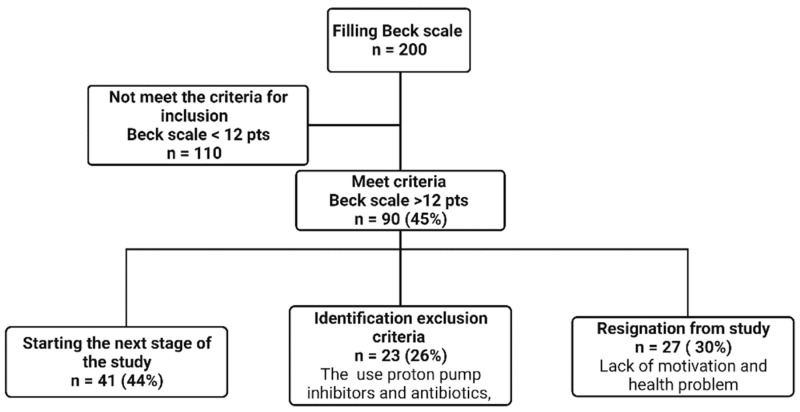
Flow chart for screening patients for further analysis. pts = points (Created with BioRender.com (accessed on 2 November 2022)).

In the period from July 2018 to December 2020, 201 patients between 18 and 70 years of age who underwent bariatric surgery (SG or RYGB method) at least 6 months earlier completed the self-assessment questionnaire Beck scale used to preselection depressive symptoms. Patients scoring ≥12 points were treated as the occurrence of depressive symptoms and included to the study [16,17,18]. Out of 91 participants who obtained ≥12 points, 27 refused to participate in this study, 23 patients were assessed with exclusion criteria (intake of antibiotics, proton pump inhibitors (PPI) and probiotics during the 6 months preceding the study as well as addiction to alcohol and psychoactive substances, internal organ failure and chronic inflammatory diseases of the gastrointestinal tract), which enabled the commencement of further tests among 41 patients (Figure 1). During the visit, biological material (blood, feces) was collected from 41 patients. Anthropometric measurements, questionnaire tests, and a food diary for the past 72 h were collected. Subsequently, in patients enrolled in the study, the severity of depressive symptoms was measured using the Hamilton scale by an experienced psychiatrist.

### 2.1. Anthropometric Research

Body composition analysis was performed using a Jawon Medical ioi-353 brand analyzer (JAWON Medical Co., Ltd., Gyeongbuk, Republic of Korea). Height (cm) was measured with the use of a measuring rod. The waist (cm) and hip circumferences (cm) were measured with a tape measure, and then the waist-hip ratio (WHR) was calculated [19].

### 2.2. Survey Research

The mental state of patients was assessed using:

- Beck’s Scale—a self-assessment questionnaire used to assess the occurrence and intensity of depressive symptoms. The total score ranges from 0 to 63 points, where the higher the score, the greater the severity of depressive symptoms. ≥12 points was considered to suggest the presence of depressive symptoms [16,17,18].
- Hamilton Scale—the questionnaire was conducted by an experienced psychiatrist to objectively assess the severity of depressive symptoms. The study used a 21-point version, where a score of ≥7 points indicated the presence of depression [20,21].
- Athenian Insomnia Scale—a scale consisting of 8 items assessing nighttime sleep patterns (falling asleep and waking up at night) as well as total sleep time, sleep quality, well-being, psychophysical fitness, and daytime sleepiness. Scores were assessed utilizing a total score of between 0 and 24 points, with a higher score representing a poorer quality of sleep. Score ≥6 points indicated insomnia [22].

### 2.3. Eating Habits

The patients’ eating habits were assessed using the food frequency questionnaire (FFQ). The questionnaire was supplemented with questions about the type of bariatric surgery, weight reduction after surgery, supplementation used, and the presence of post-operative complaints (i.e., abdominal pain, nausea, vomiting, heartburn, constipation, and diarrhea). During the visit, a food consumption list for the last 72 h was also collected and analyzed using the 5D Diet program (Food and Nutrition Institute, Warsaw). Based on the data collected with the above tools, the International Diet Quality Index (DQI-I) was calculated, with the total score ranging from 0 to 100 points. The higher the number of points, the better the quality of the diet [23].

### 2.4. Laboratory Tests

Biological material (blood, stool) was collected from the patients and then stored at −80 °C until laboratory analyses were performed. Venous blood was collected in ethylenediaminetetraacetic acid (EDTA) tubes, centrifuged (3500 rpm for 10 min), and then the plasma and morphotic parts were separated into individual Eppendorf tubes. The feces were collected using a stool sampling kit (Kałszyk, Wąchock, Poland) and then delivered by patients to the laboratory within 24 h.

### 2.5. The Assessment of Intestinal Barrier Integrity

Fecal zonulin level was determined using an enzyme-linked immunosorbent assay (ELISA) (Immundiagnostik AG, Bensheim, Germany) according to the manufacturer’s protocol. The absorbance was measured with a spectrophotometer (Sunrise, Tecan, Männedorf, Switzerland) at 450 nm. Serum lipopolysaccharide (LPS) and occludin concentrations were determined by ELISA (EIAAB Science Inc., Wuhan, China), and LPS binding protein (LBP) by ELISA (FineTest, Wuhan, China) according to manufacturers’ protocols. In each case, the absorbance was measured with a spectrophotometer (Sunrise, Tecan, Männedorf, Switzerland) at 450 nm.

### 2.6. Sequencing Analysis of the Bacterial 16S RNA Genes

DNA isolation from stools and sequencing of the V3–V4 regions of the 16S rDNA gene were performed on the Illumina MiSeq apparatus (Illumina INC, San Diego, CA, USA) at the Clinical Molecular Biology Institute of the University of Kiel (Kiel, Germany) according to their own protocol. DNA was isolated using microcentrifuge columns with silica membrane. The extracted DNA was purified using an Agencourt AMPure^®^XP machine (Beckman Coulter, Brea, CA, USA). The DNA was amplified using the Metagenomic Library Construction Kit 16S (V3–V4) for Next Generation Sequencing (Takara Bio Inc., Kusatsu, Japan), followed by sequencing using the Illumina MiSeq v3 2 × 250 bp kit (Illumina Inc., San Diego, CA, USA).

### 2.7. The Assessment of SCFA Content in Faecal Samples

The analysis was performed on 0.5 g of a faecal sample, which was then homogenized in 5 mL of water for 5 min. The pH was acidified to pH = 3 with 5M HCl and further samples were centrifuged for 20 min. The obtained samples were analyzed by gas chromatography with a flame ionization detector (FID). SCFA analysis included: acetic acid (C2: 0), propionic acid (C3: 0), isobutyric acid (C4: 0 i), butyric acid (C4: 0 n), isovaleric acid (C5: 0 i), and valeric acid (C5: 0 n). The analysis was performed using an Agilent Technologies 1260 System ( Santa Clara, USA) gas chromatograph on a DB-FFAP column, 30 m × 0.53 mm × 0.5 µm. Hydrogen was supplied as carrier gas at a flow rate of 14.4 mL/min. The starting temperature was 100 °C. It was held for 0.5 min and then raised to 180 °C at a rate of 8 °C/min and held for 1 min. The temperature was then increased to 200 °C at a rate of 20 °C/min and held at 200 °C for 5 min. The fatty acids were identified by comparing their retention times with commercially available standards.

### 2.8. Statistical Analysis

The statistical analysis was performed in the R environment (version 4.0.5) (Aucland Uniwersity, Auckland, New Zeland), and the statement of values and the graphical presentation were made in MS Excel. The groups were characterized for quantitative variables by presenting the values of the mean, median, standard deviation, minimum and maximum value, range and standard error.

Overlapping pairs of Illumina readings were analyzed using the (SanPANDAseq program (San Diego, USA) [24]. The assessment of the structure of the microbial ecosystem was based on the relative percentage of taxa. Sequences were initially identified taxonomically using the RDP 2.13 classifier (San Diego, USA) [25], then the taxonomy level i.e., genus, family, order, class, etc. was determined by assigning a level where confidence level for identification was ≥80. To find the correlation between the structure of the bacterial community and anthropometric factors, non-metric multidimensional scaling (NMDS) and redundancy analysis (RDA) from the vegan R package) (Aucland Uniwersity, Auckland, New Zeland) was used.

Sample size was determined for Permutational Analysis of Variance (PERMANOVA) which is used to detect the differences in microbial composition between two or more groups of individuals using the micropower R package for the weighted Jaccard distance metric. The necessary data for sample size estimation, i.e., within-group mean and standard deviation of the Jaccard distance, were determined using metagenomic gut samples from HMP (Human Microbiome Project). Based on the HMP dataset the smallest detectable effect size (ω2) assuming 80% was 0.0068 for 30 subjects per group (the ω2 values for various studies with statistically significant between-group differences ranged between 0.023 and 0.23) [26].

RDA was performed on Hellinger-transformed relative abundance data in four rounds until the final model was specified. The procedure was as follows: The first round was an univariate analysis. In the second round the most significant variable was tested with all other variables in pairs. Two variables from the most significant model were tested with all other variables in the round three. Three variables from the most significant model were tested in all other variables in the last round (four). The final model was the last most significant model. Non-metric multidimensional scaling was carried out using Bray-Curtis distances and non-transformed relative abundance tables. The regression of factors to the NMDS ordination axes was performed using the envfit function. To identify bacteria that are associated with factor variables we performed species indicator analysis using the multipath function from the indiscpecies package. All permutation tests were performed with 100,000 permutations. The lattice (R 3.5.1) and ggplot2 packages) (Aucland Uniwersity, Auckland, New Zeland) [27] were used to prepare the graphs. Rarefaction curves were generated using the indicspecies package) (Aucland Uniwersity, Auckland, New Zeland) [28].

## 3. Results

The mean age in the study group of 41 patients was 44.12 (±10.31) years. Even though the patients were, on average, 3 years post surgery, they still suffered from abdominal obesity. The anthropometric measurements are presented in Supplementary Table S1.

### 3.1. Mental State

The evaluation of the mental state of the patients showed the presence of mild depressive symptoms in most of them—the mean value of the Beck scale was 18.42 (±7.63) points. The presence of depressive symptoms was also confirmed by the Hamilton scale, the mean value of which was 12.94 (±4.7) points. Additionally, sleep disorders were reported among the studied patients through the Athenian Insomnia Scale (the result of which was 10.11 (±4) points). Detailed information is presented in Table 1 and Supplementary Table S1.

### 3.2. Gastrointestinal Symptoms, Endotoxaemia Parameters and Other Biochemical Results

**Figure 2:**
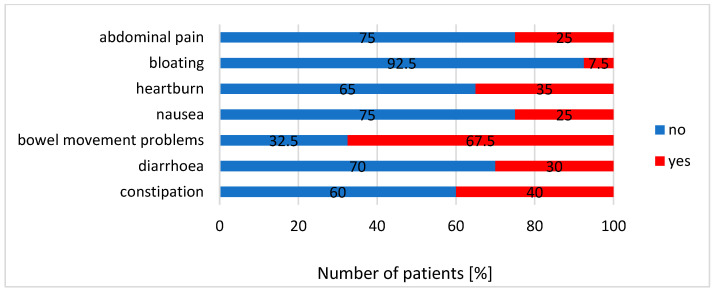
The summary of complaints reported by patients.

The most common symptom reported by patients was problems with defecation, which affected 67.5% of participants. Additionally, 40% of patients suffered from constipation and 30% had diarrhea. Flatulence (7.5%) was the least common symptom in this group of participants. A summary of all reported complaints is presented in Figure 2.

The analysis of endotoxemia parameters showed that the mean blood level of LBP in patients was 594.09 (±196.81) ng/mL, LPS 102.09 (±34.66) pg/mL, occludin 13.77 (±3.41) ng/mL, and faecal zonulin concentration 137.05 (±73.54) ng/mL. Detailed results of the performed laboratory tests are presented in Supplementary Table S1.

### 3.3. The Analysis of Diet

The analysis of participants’ diet showed that the patients consumed protein consistent with postoperative recommendations [29]. The amount of protein was 65.2 g (±16.2 g) per day. The most frequently chosen sources of protein were poultry and dairy products with a low consumption of fish and legume seeds (DQI-I diversity 9.9 (±4.72) points). Additionally, the diet of the participants was low in grains and vegetables, which translated into a low dietary fiber content—15.5 (±6.4) g (DQI-I adequacy 22.55 (±3.46) points). In addition, the patients’ diet was not satisfactory due to the relatively high consumption of SCFA (10.8% (±3.9%) of the total daily energy of the diet) and products providing so-called empty calories (mainly sweets). All these aspects contributed to the score of 47.45 (±8.32) DQI-I points (Supplementary Table S1).

### 3.4. The Composition of Gut Microbiota

**Figure 3:**
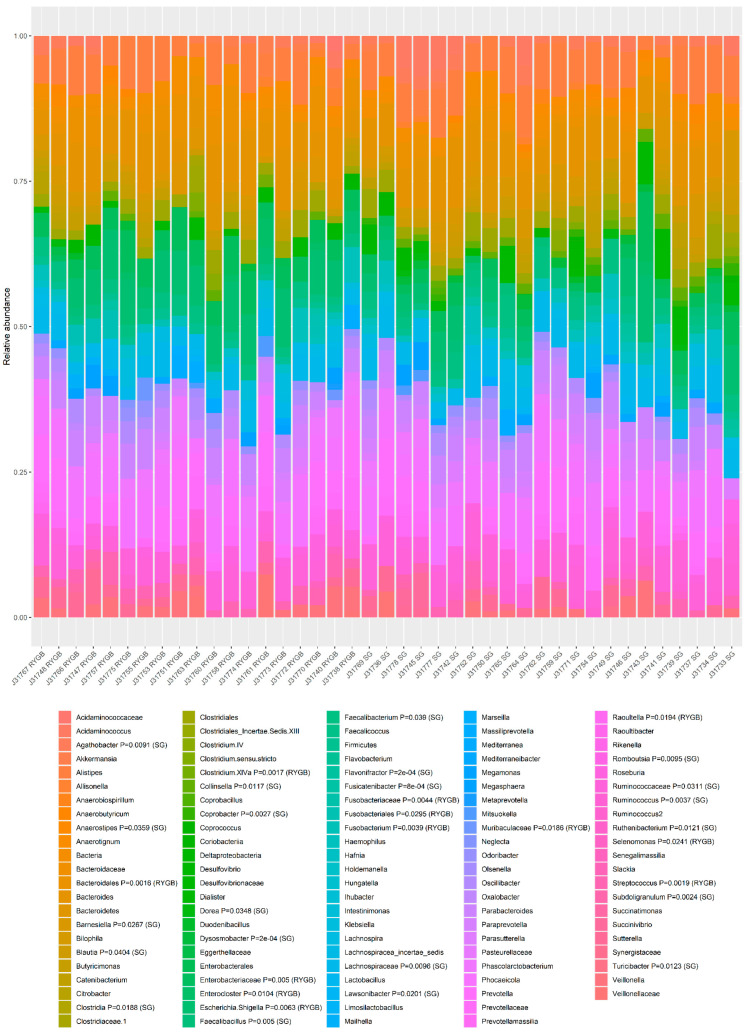
The composition of gut microbiota (relative abundance of OUT) based on the 16S rRNA gene sequencing by sample and type of surgery. Relative abundance of OTUs is shown for each patient. Patients are arranged by type of surgery. In the legend, the *p* value and group (RYGB or SG) for which the highest association was found for a given taxon are shown. The association between taxa patterns and groups was performed by multilevel pattern analysis.

The taxonomic composition of the gut microbiota obtained from all participants was expressed in terms of operational taxonomic units (OTUs) (Figure 3).

**Figure 4:**
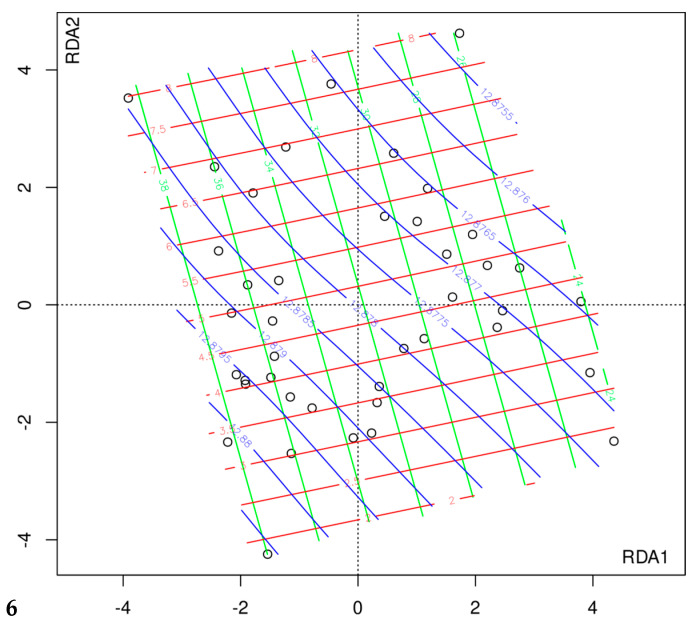
Relationship between the composition of the intestinal microbiota and Beck’s score, and the concentration of isobutyric acid and acetate in the stool. Black circles—individual patients; blue color line—Beck’s scale value; red color—the concentration of isobutyric acid in the stool; green—the concentration of acetate in the stool. The increase of the Beck sum of points (blue line) is associated with a decrease in stool isobutyric acid (red line) and an increase in stool acetate (green line).

In RDS, gut microbiota composition differed significantly among patients undergoing two different types of surgery (F = 1.64, *p* = 0.00002). Also, we found an association with short-chain fatty acids (SCFAC2, F = 1.68, *p* = 0.00003; SCFAC3, F = 1.39, *p* = 0.002; SCFAC4i, F = 1.51, *p* = 0.0003; SCFAC4n, F = 1.26, *p* = 0.020); and borderline significant association with the Beck scale (F = 1.22, *p* = 0.058). The final model was constructed after four rounds of model fitting with one, two, three, and four variables, (see Methods) and included SCFAC2, SCFAC4i, and Beck scale (Figure 4). The models tested in all rounds of variable selection process and the significance of variables are shown in Supplementary Table S2. This model suggests that the composition of the microbiota is related to the concentration of isobutyric acid and acetic acid in the stool. From the ordination plot with surfaces for each variable (Figure 4) we can conclude that a worsening of depressive symptoms is associated with a decrease in isobutyric acid concentration and an increase in faecal acetate concentration.

**Figure 5:**
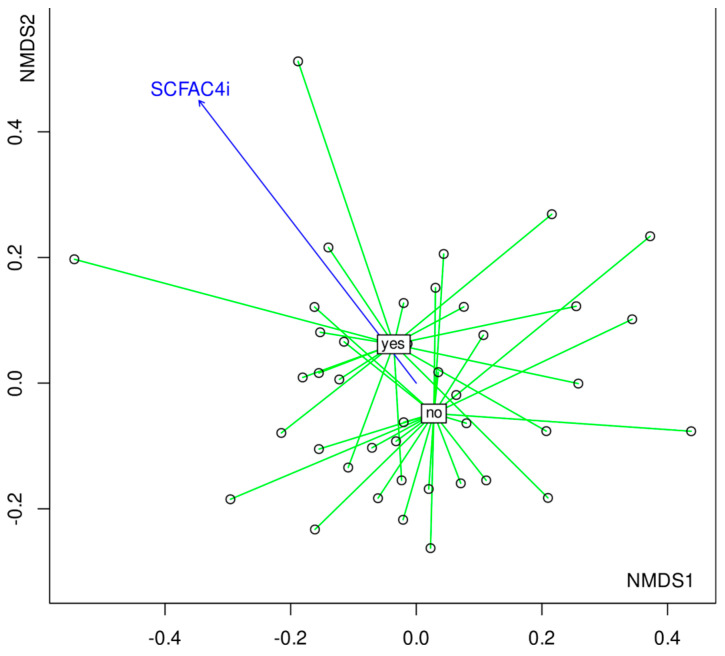
NMDS plot of bacterial communities where samples are grouped into two significantly different groups—presence/absence of gastrointestinal complaints with the arrow representing increasing concentration of isobutyric acid in the stool. Yes—the presence of gastro-intestinal symptoms; no—the lack of gastro-intestinal symptoms. The blue arrow represents direction of increasing concentration of SCFAC4i.

**Figure 6:**
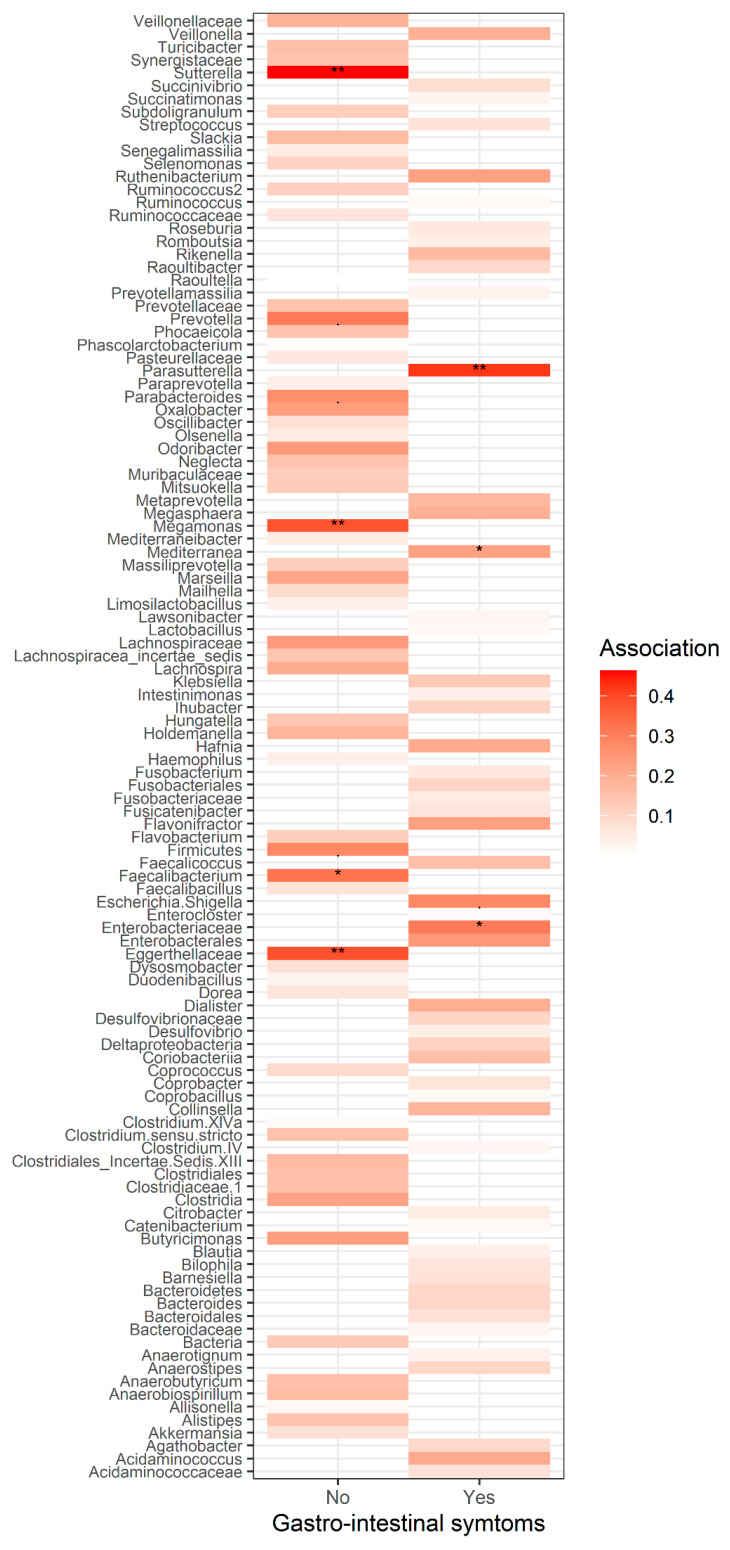
The most common taxa and the strength of association (and corresponding *p* values) with a presence/lack of gastrointestinal symptoms. * strength of association.

In addition to RDA, a non-metric multidimensional scaling (NMDS) was performed using Bray-Curtis distances calculated on relative abundances. To visualize the underlying trends in microbial composition, environmental variables were overlayed onto ordination plot by using regression of environmental variables on NMDS ordination scores (with envfit function). Two variables were significant: SCFAC4i *R*^2^ = 0.40, *p* = 0.020 and the presence of digestive symptoms (i.e., nausea, diarrhea, constipation, flatulence, and abdominal pain, *R*^2^ = 0.22, *p* = 0.0098). As these variables were co-varying with the community composition, they could possibly cause a change in the compositional pattern of this community (Figure 5). Total output of the envfit regression is shown in Supplementary Table S3. Interestingly, the presence of digestive symptoms was also associated with increased isobutyric acid levels in the feces of these patients. The examination of bacterial patterns revealed the bacteria associated with each group (Figure 5 ). Patients who did not report any complaints had significantly higher fecal content of Oxalobacteraceae, Faecalibacterium, or Megamonas (Figure 5 and Figure 6).

## 4. Discussion

To the best of our knowledge, this is the first study dedicated to investigating the relationship between the gut microbiota, the intestinal barrier markers, diet, and the occurrence of depressive symptoms among patients after surgical treatment of morbid obesity using RYGB and SG methods.

Numerous studies indicate an improvement in the mental health of patients after bariatric surgeries, including improvement of the quality of life, increased self-esteem, improved body image, or a reduction in the severity of depressive symptoms and anxiety among patients [30,31,32,33,34]. However, the results of some studies indicated a deterioration in the quality of life of patients after bariatric surgeries, which is reflected in an increase in the percentage of self-harm, depression, and alcohol abuse in this group of patients [35,36,37,38]. A 12-year cohort study showed that bariatric surgery is significantly associated with an increased risk of developing major depressive disorder. After an initial significant improvement in mental functioning, an increased risk of major depressive disorder is noted over 4 years after surgery [39]. In the study, among 200 surveyed patients (on average 3 years after bariatric surgery), as many as 45% met the criteria for the diagnosis of depressive disorders expressed by the Beck self-assessment scale. A study by Ribeiro et al. [40] showed that after the initial post-operative improvement in mental functioning (reduction of depression and anxiety symptoms in the first 23 months after RYGB), there was a gradual deterioration of the mental state of patients. Five years after the operation, depression was reported in 35% of patients, and anxiety disorders in 40% [40].

Chronic inflammation is associated with obesity. Moreover, patients with obesity are more likely to develop depression than the general population, suggesting the importance of the microbiota-gut-brain axis [41,42]. In a systematic review by Cheung et al. [43], it has been shown that the microbiota of MDD patients is depleted in microorganisms with a high ability to metabolize carbohydrates (*Bifidobacterium*, *Faecalibacterium*, and *Ruminococcus*). At the same time, the authors indicated that patients with MDD had an increased number of *Anaerostipes*, *Blautia*, and *Clostridium* (which can also metabolize carbohydrates), as well as bacteria with a high ability to metabolize proteins and amino acids (*Clostridium*, *Klebsiella*, *Parabacteroides*, *Streptococcus*, and *Oscillibacter*, *Alistipes*) [43]. Additionally, it has been shown that an increased concentration of intestinal dysbiosis markers significantly correlates with the severity of depressive symptoms. In the meta-analysis by Safadi et al. [44] it has been shown that patients suffering from depression have an increased concentration of zonulin, LPS, LBP, or alpha-1-antitrypsin, as well as a lower concentration of SCFA in the stool compared to the control group [45]. Therefore, it seems that endotoxemia may be a significant factor in connection with depression, inflammation, and obesity [41,46].

In the studied group of patients, as many as 67.5% suffered from defecation problems, 40% experienced chronic constipation, and 30% experienced chronic diarrhea. The gut microbiota of patients suffering from gastrointestinal complaints was characterized by a significantly higher relative abundance of *Enterobacteriaceae* (whose overgrowth can be seen in many inflammatory conditions, such as inflammatory bowel diseases, obesity, colorectal cancer, and celiac disease) [47], *Parasutterella* (its presence may be related to genesis and development of IBS, as well as chronic inflammation of the intestines) [48], or *Enterococcus* (opportunistic pathogens that, apart from their typical commensal environment—the gastrointestinal tract, can cause various infections). Especially *E. faecalis* and *E. faecium* are related with serious complications and nosocomial infections [49]. The presence of a significantly higher number of *Sellimonas* in the feces of this group of patients is somewhat surprising. It is postulated that these bacteria can potentially have a beneficial effect on the health of the host, and *S. intestinalis* may serve as a biomarker of the return of homeostasis within the gut microbiota [50].

The gut microbiota associated with the occurrence of gastrointestinal complaints among the studied patients was also related to the increased concentration of isobutyric acid in the feces (Figure 5). This acid, like isovaleric acid, is classified as a branched fatty acid (BCFA). They are produced by the gut microbiota mainly as a result of fermentation (mainly by *Bacteroides* and *Clostridium*) of branched-chain amino acids (valine, leucine, and isoleucine) [51]. We still know relatively little about the impact of BCFA on human health. It seems, however, that these acids can undergo oxidation if the amount of butyric acid (included in SCFA) is insufficient and constitutes a source of energy for colonocytes [52]. Additionally, an increase in the amount of BCFA in the stool may indicate an increased proteolytic fermentation (a high amount of protein in the large intestine may result from its high intake in the diet and/or malabsorption disorders) leading to the formation of harmful metabolites, i.e., ammonia, p-cresol, phenols, or hydrogen sulfide. [51,53,54]. This, in turn, may contribute to the disturbance of the structure of the colon epithelium and the development of inflammation of the mucosa. It may also affect the intestinal nervous system and intestinal motility. This is of significant clinical importance in inflammatory bowel diseases or colorectal neoplasms [55,56]. Additionally, it has been shown that BCFA (isobutyric and isovaleric acid) may be one of the factors contributing to the development of depressive disorders [57]. In studies conducted in patients after bariatric surgery, an increase in the concentration of BCFA in the feces as well as a decrease in the SCFA/BCFA ratio and a negative correlation between the consumption of starch and the concentration of BCFA in the feces have been reported [58,59,60,61]. Therefore, in order to limit proteolytic fermentation, it seems justified to pay special attention to the appropriate dietary fiber intake in the diet of patients after bariatric surgery [56,60]. In the study, the patients consumed the recommended amount of protein, mainly from poultry and dairy products (DQI-I diversity 9.9 (±4.72) points), with a simultaneous low consumption of cereal products and fiber (DQI-I adequacy 22.55 (±3.46) points), which could have influence on a significant increase in the concentration of isobutyric acid in the stool of people suffering from gastrointestinal ailments (Figure 6). Also, in other studies [62,63], a low consumption of fiber is reported in the bariatric population, which undoubtedly has a negative impact on the functioning of the body [64].

In the current study, the normal BMI range was observed only in 6 patients and more than 50% of patients suffered from obesity (see Supplementary Table S4). Obesity is characterized by low-level inflammation, which contributes to the development of many comorbidities [65]. Studies have shown that the concentration of LPS is higher in obese people than in lean people, and by unsealing the intestinal barrier, it can get into the circulatory system [66]. Obviously, small amounts of LPS (as a component of the cell membrane of gram-negative bacteria) also enter the circulation in healthy people, but its increased translocation occurs after eating high-fat meals [67,68,69,70,71]. In particular, saturated fatty acids may promote low-intensity chronic inflammation and induce an increase in LPS levels [66,72]. Interestingly, in a study focusing on the analysis of the microbiota of patients who experienced a re-gain in body weight after bariatric surgery, it was shown that they are characterized by lower microbiota abundance, and also have lower numbers of *Sarcina*, *Butyrivibrio*, *Alkaliphilus*, *Lachnospira*, *Pseudoalteromonas*, and *Cetobacterium* in relation to people who have achieved successful weight loss after surgery [73].

### Limitations

This study has several limitations worth noting. Although we stated that the number of patients who met the inclusion criteria were 91, only 41 people were enrolled in this research. Different surgery methods (SG and RYGB) and the obesity of most participants can be a significant confounder factor of microbiota analysis. This undoubtedly makes it necessary to repeat the study on a larger group of participants. Secondly, it is worth remembering that BCFA in the stool is dependent on their synthesis, utilization, and absorption. In this study, we examined only BCFA level in the stool (without blood level concentration).

## 5. Conclusions

The type of bariatric surgery affects the microbiota in different ways. Studies have suggested that the gut microbiota may play a role in the development of depressive symptoms and gastrointestinal complications in patients after bariatric surgery. Low fiber consumption and an increased level of fecal isobutyric acid may indicate increased proteolytic fermentation and lead to intestinal inflammation. There is a need for further research on this topic, including a bigger sample size.

## Figures and Tables

**Table 1:** Individual patients characteristics.

UPN	Age	Gender	Type of Surgery	Months after Surgery	Beck’s Scale	Hamilton’s Scale	Athenian Insomnia Scale
J31767	47	Woman	RYGB	60	16	11	8
J31748	47	Woman	RYGB	60	12	11	10
J31766	59	Woman	RYGB	60	2	2	5
J31747	59	Woman	RYGB	60	12	12	10
J31757	60	Woman	RYGB	48	14	NA	NA
J31775	45	Woman	RYGB	6	2	NA	NA
J31755	45	Woman	RYGB	6	12	NA	NA
J31753	48	Woman	RYGB	19	18	14	10
J31751	38	Woman	RYGB	49	3	10	7
J31763	30	Woman	RYGB	25	6	NA	NA
J31760	54	Woman	RYGB	145	16	17	11
J31758	38	Woman	RYGB	20	10	10	12
J31774	52	Woman	RYGB	8	9	7	11
J31761	48	Woman	RYGB	13	7	4	3
J31773	48	Woman	RYGB	25	6	4	4
J31772	41	Woman	RYGB	109	9	NA	NA
J31770	64	Woman	RYGB	7	14	19	3
J31740	51	Woman	RYGB	9	29	17	11
J31738	38	Woman	RYGB	120	19	19	6
J31769	45	Woman	SG	30	9	0	0
J31736	45	Woman	SG	30	12	NA	NA
J31778	36	Woman	SG	11	5	NA	NA
J31745	36	Woman	SG	11	23	15	11
J31777	34	Woman	SG	9	4	8	7
J31742	34	Woman	SG	9	12	13	6
J31752	28	Woman	SG	14	5	9	6
J31750	55	Woman	SG	15	2	2	6
J31765	23	Woman	SG	18	22	6	14
J31764	48	Woman	SG	15	18	7	11
J31762	49	Woman	SG	61	11	10	10
J31759	44	Woman	SG	61	32	21	11
J31771	66	Woman	SG	109	3	NA	NA
J31754	36	Man	SG	24	12	NA	NA
J31749	31	Man	SG	18	21	13	10
J31746	36	Woman	SG	48	12	9	9
J31743	28	Woman	SG	6	21	12	13
J31741	50	Woman	SG	48	12	NA	NA
J31739	47	Woman	SG	14	34	19	18
J31737	35	Man	SG	15	18	7	6
J31734	49	Woman	SG	12	12	9	13
J31733	44	Woman	SG	9	12	7	11

UPN—Unique patient name; RYGB—Roux-en-Y gastric bypass, SG—sleeve gastrectomy; NA—not available.

## Data Availability

Not applicable.

## Supplementary Materials

The following supporting information can be downloaded at: https://www.mdpi.com/article/10.3390/nu14245372/s1, Table S1: Descriptive statistics of all variables used in the study; Table S2: Steps of the RDA analysis–variable selectin process; Table S3: The coefficients of determination (*R*^2^) and *p* values from regression analysis of variables (vectors or factors) with nMDS ordination scores using envfit function (vegan package); Table S4: BMI of patients enrolled in the study.
