# Supplementary material for: Association between Fecal Microbiota, SCFA, Gut Integrity Markers and Depressive Symptoms in Patients Treated in the Past with Bariatric Surgery—The Cross-Sectional Study
Source: Nutrients. 2022 Dec 17;14(24):5372. doi: 10.3390/nu14245372 (PMC9781380; doi:10.3390/nu14245372)
Supplement: Supplementary file 1 [file nutrients-14-05372-s001.zip › nutrients-2083263-supplementary.pdf]

nutrients

Supplementary Table S1. Descriptive statistics of all variables used in the study.

Parameters	Mean	SD	Median	Min	Max	Range	Skewness	Kurtosis	SE
Waist [cm]	97.86	12.82	97.5	71	122	51	−0.12	−0.89	2.03
WHR	0.86	0.09	0.85	0.67	1.06	0.39	0.27	−0.17	0.01
Body mass [kg]	88.02	17.4	86.6	62.1	125.7	63.6	0.52	−0.78	2.75
FFM [kg]	58.05	10.08	56.8	44.7	88.2	43.5	0.83	0.25	1.59
FM [kg]	30.66	10.1	29.05	14.1	54.1	40	0.52	−0.43	1.6
BMI [kg/m²]	30.66	5.13	30.3	20	40.5	20.5	0.22	−0.62	0.81
Beck's scale	18.42	7.63	17	12	44	32	1.47	2.01	1.21
Hamilton's scale	12.94	4.7	13	5	24	19	0.13	−0.66	0.79
Athenian insomnia scale	10.11	4	10	1	19	18	0.01	−0.24	0.68
LBP [ng/ml]	594.09	196.81	528.12	363.98	1467.2	1103.22	2.49	7.79	31.12
LPS [pg/ml]	102.09	34.66	97.84	55.29	206.64	151.35	0.93	0.42	5.48
Fecal zonulin [ng/ml]	137.05	73.54	119.29	37.79	345.06	307.27	0.86	0.2	13.66
Occludin [ng/ml]	13.77	3.41	12.94	6.5	25.52	19.02	1.07	1.87	0.54
C 2:0	33.01	5.25	32.44	21.8	43.6	21.8	0.15	−0.74	0.85
C 3:0	21.57	5.79	21.86	10.53	39.29	28.76	0.36	0.56	0.94
C 4:0i	4.74	1.71	4.49	2.11	8.33	6.23	0.44	−0.82	0.28
C 4:0n	22.75	7.06	22.75	10.15	39.92	27.77	−0.07	−0.95	1.15
C 5:0i	8.96	3.87	8.47	3.16	18.63	15.47	0.68	−0.4	0.63
C 5:0n	6.09	1.9	6.4	0.85	9.59	8.75	−0.79	0.93	0.31
DQI−I	47.45	8.32	47	35	71	36	0.56	−0.05	1.32
Diversity	9.9	4.72	10	3	20	17	0.25	−0.59	0.75
Adequacy	22.55	3.46	23	15	29	14	−0.25	−0.73	0.55
Moderation	14.4	4.52	15	6	24	18	−0.46	−0.81	0.71
Overall diet balance	0.6	1.45	0	0	8	8	3.47	14.32	0.23

SD – standard deviation, SE – standard error, Range – Max – Min.

Supplementary Table S2. Steps of the RDA analysis – variable selectin process.

Round 1.			Round 2			Round 3			Round 4		
	F	P	SCFAC2 +	F	P	SCFAC2 + SCFAC4i +	F	P	SCFAC2 + SCFAC4i + Beck scale +	F	P
Surgery type	1.6356	5.00E−05	Surgery type	1.1062	0.1881	Surgery type	1.1163	0.16891	Surgery type	1.1178	0.16523
Digestive symptoms	1.0316	0.3342	Digestive symptoms	1.0497	0.2733	Digestive symptoms	1.0375	0.3128	Digestive symptoms	0.9968	0.47673
Abdominal pain	1.0543	0.2858	Abdominal pain	1.1198	0.1471	Abdominal pain	1.1228	0.14283	Abdominal pain	1.077	0.23912
Bloating	1.0654	0.3476	Bloating	1.0113	0.4412	Bloating	1.0125	0.4398	Bloating	0.9911	0.47833
Heart burn	1.0215	0.3933	Heart burn	1.0595	0.2915	Heart burn	1.0665	0.2753	Heart burn	1.1039	0.1964
Nausea	0.839	0.8728	Nausea	0.8636	0.8227	Nausea	0.8633	0.8254	Nausea	0.8503	0.8544

Defecation problem	1.0215	0.3726	Defecation problem	0.9777	0.5597	Defecation problem	0.9835	0.535	Defecation problem	0.9627	0.61931
Diarrhoea	0.9778	0.5391	Diarrhoea	0.993	0.4865	Diarrhoea	0.9675	0.5752	Diarrhoea	0.9496	0.64024
Constipation	1.1728	0.07281	Constipation	1.0961	0.1878	Constipation	1.1026	0.17574	Constipation	1.1088	0.1639
Age	1.0876	0.2211	Age	1.0286	0.3784	Age	0.9867	0.51949	Age	0.9866	0.52182
Beck scale	1.2185	0.05855	Beck scale	1.1986	0.06947	**Beck scale**	**1.2235**	**0.05585**			
BMI	0.9947	0.4904	BMI	1.0007	0.4693	BMI	0.96	0.60522	BMI	0.9619	0.60175
Months after surgery	1.0339	0.3742	Months after surgery	1.0495	0.3349	Months after surgery	1.0496	0.33277	Months after surgery	1.0496	0.33272
LBP	1.0352	0.3608	LBP	1.0442	0.3357	LBP	1.0596	0.29357	LBP	1.0404	0.34611
LPS	0.9558	0.634	LPS	0.9645	0.5992	LPS	0.9768	0.55337	LPS	0.9912	0.50366
Homocysteine	1.1052	0.1946	Homocysteine	1.0823	0.2358	Homocysteine	0.9762	0.5439	Homocysteine	0.9732	0.55292
Occludin	1.0314	0.3791	Occludin	1.0247	0.3974	Occludin	1.0368	0.36497	Occludin	1.0456	0.33783
Vitamin D	1.0476	0.3198	Vitamin D	1.0672	0.2632	Vitamin D	1.1556	0.08927	Vitamin D	1.1636	0.08147
Waist	1.1209	0.141	Waist	1.1318	0.1249	Waist	1.0674	0.2679	Waist	1.0743	0.24952
WHR	1.1109	0.1743	WHR	1.0978	0.1984	WHR	1.0771	0.2434	WHR	1.0859	0.22391
Weight	1.0641	0.2698	Weight	1.0688	0.2605	Weight	1.002	0.46053	Weight	1.0086	0.44286
LBM	1.1702	0.1163	LBM	1.1447	0.1484	LBM	1.0784	0.2514	LBM	1.0892	0.22809
Fat mass	0.954	0.6351	Fat mass	0.9757	0.5573	Fat mass	0.945	0.65729	Fat mass	0.9506	0.63922
**SCFAC2**	**1.6829**	**2.00E−05**									
SCFAC3	1.3945	0.00165	SCFAC3	1.1881	0.06222	SCFAC3			SCFAC3	1.1137	0.14736
SCFAC4i	1.5113	0.00026	**SCFAC4i**	**1.5466**	**0.00015**		1.0892	0.2009			
SCFAC4n	1.2597	0.02084	SCFAC4n	1.1091	0.1756	SCFAC4n	0.9604	0.6185	SCFAC4n	0.9906	0.5062
SCFAC5n	1.1556	0.1218	SCFAC5n	1.2143	0.0608	SCFAC5n	1.2558	0.05656	SCFAC5n	1.2622	0.05438

.

Supplementary Table S3. The coefficients of determination (R^2) and P values from regression analysis of variables (vectors or factors) with nMDS ordination scores using envfit function (vegan package).

**Variable (Vectors or Factors)**	R^2	*p*
Age	0.0156	0.88954
Beck scale	0.0044	0.9688
Waist	0.0116	0.91805
WHR	0.0517	0.67083
Weight	0.0256	0.8282
LBM	0.0297	0.80112
Fat mass	0.0237	0.84085
BMI	0.047	0.70326
Months after surgery	0.008	0.93631
LBP	0.0477	0.69721
LPS	0.3086	0.05812
Homocysteine	0.1261	0.3662
Zonulin	0.28	0.08667
Occludin	0.1644	0.25871
Vitamin D	0.0881	0.50351
SCFAC2	0.1717	0.24236
SCFAC3	0.1776	0.23172

SCFAC4i	0.4047	0.01935
SCFAC4n	0.4046	0.01989
SCFAC5i	0.2614	0.10216
SCFAC5n	0.0642	0.59377
Surgery type	0.0546	0.42767
Digestive symptoms	0.2243	0.00986
Abdominal pain	0.0806	0.25485
Bloating	0.125	0.16489
Heart burn	0.1335	0.09068
Nausea	0.046	0.38899
Defecation problem	0.0867	0.24193
Diarrhoea	0.04	0.54347
Constipation	0.0533	0.41512

.

Supplementary Table S4. BMI of patients enrolled in the study.

**BMI Range (kg/m²)**	**Number of Patients**
BMI <18.5	0
BMI ≥ 18.5 & BMI <25	6
BMI ≥ 25 & BMI <30	13
BMI ≥30	22
BMI ≥30 & BMI <35	13
BMI ≥35 & BMI <40	6
BMI ≥40	3
